# The analysis of a new fractional model to the Zika virus infection with mutant

**DOI:** 10.1016/j.heliyon.2023.e23390

**Published:** 2023-12-09

**Authors:** Zain Ul Abadin Zafar, Muhammad Altaf Khan, Mustafa Inc, Ali Akgül, Mohammed Asiri, Muhammad Bilal Riaz

**Affiliations:** a Department of Mathematics, Faculty of Science and Technology, University of Central Punjab, Lahore, Pakistan; b Institute for Ground Water Studies, Faculty of Natural and Agriculture Sciences, University of the Free State, South Africa; c Department of Mathematics, Science Faculty, Firat University, Elazig, Turkey; d Department of Medical Research, China Medical University, Taichung, Taiwan; e Department of Computer Science and Mathematics, Lebanese American University, Beirut, Lebanon; f Siirt University, Art and Science Faculty, Department of Mathematics, 56100 Siirt, Turkey; g Near East University, Mathematics Research Center, Department of Mathematics, Near East Boulevard, PC: 99138, Nicosia, Mersin 10, Turkey; h Department of Clinical Laboratory Sciences, College of Applied Medical Sciences, King Khalid University, P.O. Box 61413, Abha 9088, Saudi Arabia; i IT4Innovations, VSB – Technical University of Ostrava, Ostrava, Czech Republic; j Department of Computer Science and Mathematics, Lebanese American University, Byblos, Lebanon; k Department of Computer Engineering, Biruni University, 34010 Istanbul, Turkiye

**Keywords:** Zika model with mutant, AB operator, Simulation results, Existence and uniqueness, Sensitivity analysis

## Abstract

We present a new mathematical model to analyze the dynamics of the Zika virus (ZV) disease with the mutant under the real confirmed cases in Colombia. We give the formulation of the model initially in integer order derivative and then extend it to a fractional order system in the sense of the Mittag-Leffler kernel. We study the properties of the model in the Mittag-Leffler kernel and establish the result. The basic reproduction of the fractional system is computed. The equilibrium points of the Zika virus model are obtained and found that the endemic equilibria exist when the threshold is greater than unity. Further, we show that the model does not possess the backward bifurcation phenomenon. The numerical procedure to solve the problem using the Atangana-Baleanu derivative is shown using the newly established numerical scheme. We consider the real cases of the Zika virus in Colombia outbreak are considered and simulate the model using the nonlinear least square curve fit and computed the basic reproduction number $R_{0}=0.4942\text{,}$ whereas in previous work (Alzahrani et al., 2021) [1], the authors computed the basic reproduction number $R_{0}=0.5447$. This is due to the fact that our work in the present paper provides better fitting to the data when using the fractional order model, and indeed the result regarding the data fitting using the fractional model is better than integer order model. We give a sensitivity analysis of the parameters involved in the basic reproduction number and show them graphically. The results obtained through the present numerical method converge to its equilibrium for the fractional order, indicating the proposed scheme's reliability.

## 1 Introduction

Zika virus infection or disease is related to the family *Flaviviridae* with same as some other diseases including Japanese encephalitis, yellow fever, dengue and so on. Mosquitoes are responsible for the disease transmission to humans by the bite of female mosquito. Besides transmission, sexual transmission also exists for the ZV disease. Due to ZV there are so many outbreaks across the countries of the world, such as Brazil, Honduras, Canada, El Salvador, Venezuela etc. With so many infected cases. Countries including, France, Canada, Spain, Italy etc., where the infection cases are recorded [1,2].

In literature numerous research work are published in order to identify the ZV infection (ZVI) and its possible elimination from the society. For example, the authors studied the real data for 2007, and 2013–2014 data respectively for Island and Polynesia [3]. The data of disease surveillance are considered in Ref. [4]. The analysis of ZVI through an SIR model is studied in Ref. [5]. The ZI with other diseases is reported in Ref. [6]. The control analysis modeling for ZI is considered at [7]. In Ref. [8] researchers considered ZI via sexual transmission. A ZV system with actual cases from Colombia was concerned in Ref. [9].

The modeling of infectious diseases though fractional derivatives is considered frequently now-a-days due to the disease previous history. The importance of the fractional derivative to the disease modeling with memory and heredity properties and the best fitting results make it more important than the integer order modeling. In literature, there are many mathematical models were considered to study infectious illnesses. Using the fractional derivative, the authors formulated a mathematical model for coronavirus infection in Ref. [10]. The TB infection modeling with real cases from Khyber Pakhtunkhwa Pakistan is studied in fractional order model in Ref. [11]. Modeling the Hepatitis E dynamics thorough fractional model is considered in Ref. [12]. The Ebola virus with different fractional operators is considered in Ref. [13]. The application of fractional derivative to Pine wild disease is studied in Ref. [14]. The nCoV-2019 infection and its modeling in fractional derivatives, we suggest [[15], [16], [17], [18], [19], [20]]. For the SIQ dynamical COVID-19 system, the authors of [21] used artificial neural networks Levenberg-Marquardt back propagation. Paper has shown both theoretical and numerical processes. The Caputo Fabrizio fractional order model for the dynamics of the COVID-19 model with two strains is approximatively solved using the two-step fractional Adams-Bashforth technique in Ref. [22]. A fractional order technique is used in Ref. [23] to evaluate the effectiveness of face masks in preventing the spread of COVID-19 in the population. The Euler, RK-2, and RK-4 schemes were utilized by Salihu S. Musa et al. to demonstrate the model's physical characteristics and produce approximative solutions [24]. New COVID-19 transmission was controlled optically in accordance with [25]. The authors employed three different methodologies, namely the Euler, RK-2, and RK-4 to analyze and evaluate the impact of lock-down on the dynamics of COVID-19 in Nigeria [26].

[27] examined the efficacy of the methods used by decision-makers to stop the spread of COVID-19. Although the same author et al. [28] used the COVID-19 model to study the dynamics of disease dissemination in Nigeria, Baba et al. [29] examined fractional order COVID-19 model theoretically and numerically using Caputo operator. The Caputo fractional operator was employed by the authors of [30] to examine the COVID-19 with lockdown.

In the present paper we aim to study ZV model with mutant using AB derivative. We consider actual cases of ZV and get parameters. Parameters obtained through fitting are used further to simulate the model for the equilibriums. We provide more suitable fitting with the proposed computational method to solution of the model. We organize rest of the paper as: Fundamental fractional concepts considered in the modeling of this paper are shown in section 2. We show formulations by AB derivative in the 3rd section. Section 4 discusses the equilibrium points and its analysis, and the estimations of the model parameters. Existence-uniqueness, and approximate scheme for system are shown respectively in parts 5 and 6. Section 7 discusses the numerical scheme for the given model. The sensitivity analysis is shown in section 8. Results and discussion are shown in section 9 and concluding remarks in section 10.

## 2 Fundamentals

The Liouville-Caputo operator is written [31] as:

(1) Dtτ1,τ20FFC[h(t)]=1Γ(n−τ1)∫0td[h(ζ)]dtτ2(t−ζ)n−τ1−1dζ,n−1<τ1,τ2≤n∈N,

in which

(2) $$\frac{d\;\left[h\left(\zeta \right)\right]}{dt^{\tau_{2}}}=\lim_{t\to \zeta }\frac{h\left(t\right)-h\left(\zeta \right)}{t^{\tau_{2}}-\zeta^{\tau_{2}}}\;\text{.}$$

Equation (2) is the fractal derivative definition.

As an essential special example, for $\tau_{2}=1$, Eq. (1) is reduced to Liouvelle-Caputo (LC) derivative Dtτ0C set as under [32]:

Dtτ10LC[h(t)]=1Γ(n−τ1)∫0td[h(ζ)]dt(t−ζ)n−τ1−1dζ,n−1<τ1≤n∈N,

A new important Atangana-Baleanu Caputo type fractal fractional derivative (FFABC) was studied in Ref. [31]:

(3) Dtτ1,τ20FFABC[h(t)]=Z(τ1)Γ(n−τ1)∫0td[h(ζ)]dtτ2Eτ1(τ1(t−ζ)τ1−nτ1)dζ,n−1<τ1,τ2≤n∈N,

where,

$$\frac{d\;\left[h\left(\zeta \right)\right]}{dt^{\tau_{2}}}=\lim_{t\to \zeta }\frac{h\left(t\right)-h\left(\zeta \right)}{t^{\tau_{2}}-\zeta^{\tau_{2}}}\text{.}$$

As a vital exceptional case for $\tau_{2}=1$, Eq. (3) maps to the renowned ABC non-integer derivative set by [33]

Dtτ10ABC[h(t)]=Z(τ1)Γ(n−τ1)∫0td[h(ζ)]dtEτ1(τ1(t−ζ)τ1−nτ1)dζ,n−1<τ1≤n∈N,

in which $Z\left(\tau_{1}\right)$ is a function known as normalization, Z(1)=Z(0)=1. It may be observed that the Mittag-Leffler fraction may be applied. It will conclude the description of non-local and non-singular kernel chattels, too.

Liouvelle-Caputo non-integer integral for the given function of h(t) can be described as shadows:

Itτ10LC[h(t)]=h(t)−h(0)=1Γ(τ1)∫0th(ζ)(t−ζ)τ1−1dζ.

The analogous demarcation to ABC non-integer integral for h(t) is given below [33]:

Itτ10ABC[h(t)]=h(t)−h(0)=1−τ1Z(τ1)h(t)+τ1Γ(τ1)Z(τ1)∫0th(ζ)(t−ζ)τ1−1dζ.

## 3 Mathematical model

The mathematical exemplary of ZV model [1] is given below:

(4) $$\left\{ \begin{array}{c} D_{t}S_{h}\left(t\right)={\;E}_{h}\Lambda_{H}+\left(\varphi +\Lambda_{H}\right)\left(1-E_{h}-S_{h}-I_{h}\right)-\beta_{h}^{\prime }S_{h}I_{m},\\ \begin{array}{c} D_{t}E_{h}\left(t\right)=\beta_{h}^{\prime }S_{h}I_{m}-\left(\Theta_{H}+\Psi_{H}\right)E_{h},\\ \begin{array}{c} D_{t}I_{h}\left(t\right)=\Theta_{H}E_{h}-\kappa_{H}I_{h},\\ D_{t}E_{m}\left(t\right)=\beta_{M}\left(1-I_{m}-E_{m}\right)-\left(\Theta_{M}+\Psi_{M}\right)E_{m}, \end{array} \end{array}\\ D_{t}I_{m}\left(t\right)=\Theta_{M}E_{m}-\Psi_{M}I_{m}.\; \end{array}\right.$$

The formulation of fractional Zika model (4) is in ABC sense given below

(5) {Dtτ0ABCSh(t)=EhΛH+(ΛH+φ)(1−Eh−Sh−Ih)−βh′ShIm,Dtτ0ABCEh(t)=βh′ShIm−(ΘH+ΨH)Eh,Dtτ0ABCIh(t)=EhΘH−IhκH,Dtτ0ABCEm(t)=βM(1−Em−Im)−(ΘM+ΨM)Em,Dtτ0ABCIm(t)=ΘMEm−ΨMIm.

where 0<τ<1, is the non-integer order, and IC in (5):

$$S_{h}\left(0\right)=c_{1},E_{h}\left(0\right)=c_{2},I_{h}\left(0\right)=c_{3},E_{m}\left(0\right)=c_{4},I_{m}\left(0\right)=c_{5}\text{.}$$

We give the biologically feasible region our proposed model (5) is $\Sigma =\left\{\left(S_{h},E_{h},I_{h},E_{m},I_{m}\right)|0\leq S_{h}\leq 1-E_{h}-I_{h},0\leq I_{m}\leq 1-E_{m}\right\}$ .

## 4 Equilibrium points and threshold parameter

Here, we are going to determine the number called basic reproduction $R_{0}$ for system (4) follows method in Refs. [25,34], and getting:

$F=\left(\begin{array}{ccc} 0 & 0 & \begin{array}{cc} 0 & \beta_{h}^{\prime } \end{array}\\ 0 & 0 & \begin{array}{cc} 0 & 0 \end{array}\\ \begin{array}{c} 0\\ 0 \end{array} & \begin{array}{c} \beta_{M}\\ 0 \end{array} & \begin{array}{cc} \begin{array}{c} 0\\ 0 \end{array} & \begin{array}{c} 0\\ 0 \end{array} \end{array} \end{array}\right)$ and $V=\left(\begin{array}{ccc} \Theta_{H}+\Psi_{H} & 0 & \begin{array}{cc} 0 & \;0 \end{array}\\ {-\Theta }_{H} & \kappa_{H} & \begin{array}{cc} 0 & \;0 \end{array}\\ \begin{array}{c} 0\\ 0 \end{array} & \begin{array}{c} 0\\ 0 \end{array} & \begin{array}{cc} \begin{array}{c} \Theta_{M}+\Psi_{M}\\ {-\Theta }_{M}\; \end{array} & \begin{array}{c} 0\\ \Psi_{M} \end{array} \end{array} \end{array}\right)$.with ρ(F/V), we get

$$R_{0}=\sqrt{\frac{\beta_{h}^{\prime }\Theta_{H}\Theta_{M}\beta_{M}}{\kappa_{H}\Psi_{M}\left(\Theta_{H}+\Psi_{H}\right)\left(\Theta_{M}+\Psi_{M}\right)}}$$

If the threshold parameter $R_{0}<1$, then illness stops being exists whereas for $R_{0}>1$, the disease keeps being exists.

### 4.1 We get the following system from (5) by letting

(6) {Dtτ0ABCSh(t)=0=ΛHEh+(ΛH+φ)(1−Sh−Eh−Ih)−βh′ShIm,Dtτ0ABCEh(t)=0=βh′ShIm−(ΘH+ΨH)Eh,Dtτ0ABCIh(t)=0=ΘHEh−κHIh,Dtτ0ABCEm(t)=0=βM(1−Em−Im)−(ΘM+ΨM)Em,Dtτ0ABCIm(t)=0=ΘMEm−ΨMIm.

Two equilibrium points of system (6) are obtained, i.e.,

(7) $$\left\{ \begin{array}{c} E_{0}=\left(1,0,0,0,0\right),\\ E_{1}=\left(S_{h*},E_{h*},I_{h*},E_{m*},I_{m*}\right), \end{array}\right.$$

where $S_{h*}=\frac{\kappa_{H}\left(\Theta_{H}+\Psi_{H}\right)I_{h*}}{\beta_{h}^{\prime }\Theta_{H}I_{m*}}$, $E_{h*}=\frac{\kappa_{HI_{h*}}}{\Theta_{H}}$,

$$E_{m*}=\frac{\beta_{M}\Psi_{M}I_{h*}}{\left(\Theta_{M}+\Psi_{M}\right)\left(I_{h*}\beta_{M}+\Psi_{M}\right)},I_{m*}=\frac{\beta_{M}\Theta_{M}I_{h*}}{\left(\Theta_{M}+\Psi_{M}\right)\left(I_{h*}\beta_{M}+\Psi_{M}\right)}\text{,}$$

We obtain:

(8) $$\xi_{1}I_{h*}+\xi_{2}=0\text{,}$$

in which equation (8) has the following representations for $\xi_{1}$ and $\xi_{2}$,

$$\left\{ \begin{array}{c} \xi_{1}=\beta_{M}\left(\beta_{h}^{\prime }\Theta_{M}\left(\Theta_{H}\left(\Lambda_{H}+\varphi +\kappa_{H}\right)+\kappa_{H}\left(\varphi +\Psi_{H}\right)\right)+\kappa_{H}\left(\Theta_{H}+\Psi_{H}\right)\left(\Lambda_{H}+\varphi \right)\left(\Psi_{M}+\Theta_{M}\right)\right),\\ \xi_{2}=\left(\varphi +\Lambda_{H}\right)\left(\Psi_{M}\kappa_{H}\left(\Psi_{H}+\Theta_{H}\right)\left(\Psi_{M}+\Theta_{M}\right)-\beta_{h}^{\prime }\Theta_{H}\Theta_{M}\beta_{M}\right).\; \end{array}\right.$$

We get a unique EE as equation (8) has positive coefficients.

### 4.2 Parameter estimation

**Fig. 1:**
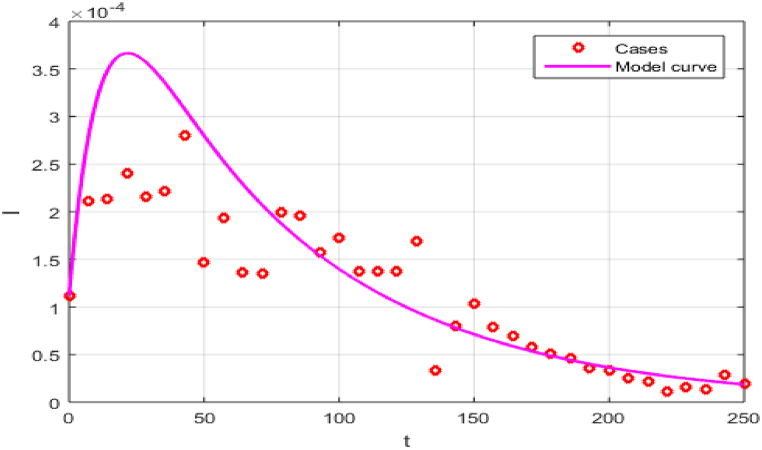
Model versus data fitting. Bold line denotes the model solution while circle represents the real data.

We first fit the model parameters to the real data considered and used in Ref. [1] for ZV model. In order to obtain data fitting for the infected data of ZV in Colombia 2016, we consider the parameter values $\frac{1}{\Theta_{M}}=10\;days$ [20] and is $\frac{1}{\Psi_{M}}=15$ days while other parameters are suitable to model. Population of Colombia is considered to be during the outbreak year is 19,471,223. Using the proportions of infected $I_{h}\left(0\right)$ , we can obtain IC to proposed model. We can get initial value for susceptible humans by considering $S_{h}\left(0\right)$ $=1\;-\;E_{h}\left(0\right)\;-\;R_{h}\left(0\right)-\;I_{h}\left(0\right)$, and with assumptions $I_{h}\left(0\right)=R_{h}\left(0\right)$. We set the initial conditions for the mosquitoes' compartments using the data fitting, the reason is the non-availability of the real data for mosquito's populations. After simulation the model using the nonlinear least square curve fitting techniques, we obtain the model parameters and the desired fitting. The data fit well to the proposed system (5) for τ=1, with $R_{0}=0.4942<\;1$, and $\beta_{h}=0.5689$, see Fig. 1, and the rest of the parameters are shown under discussion and outcomes.

## 5 Solution for (5)

Make use of the non-integer integral operator of AB in Eq. (5), we shall have

(9) $$\left\{ \begin{array}{c} S_{h}\left(t\right)-S_{h}\left(0\right)=\frac{1-\tau }{Z\left(\tau \right)}\left(\Lambda_{H}{\;E}_{h}\left(t\right)+\left(\Lambda_{H}+\varphi \right)\left(1-S_{h}\left(t\right)-E_{h}\left(t\right)-I_{h}\left(t\right)\right)-\beta_{h}^{\prime }S_{h}{\left(t\right)I}_{m}\left(t\right)\right)+\\ \frac{\tau }{Z\left(\tau \right)\Gamma \left(\tau \right)}\int_{0}^{t}{\left(t-\zeta \right)}^{\tau -1}\left(\Lambda_{H}{\;E}_{h}\left(\zeta \right)+\left(\Lambda_{H}+\varphi \right)\left(1-S_{h}\left(\zeta \right)-E_{h}\left(\zeta \right)-I_{h}\left(\zeta \right)\right)-\beta_{h}^{\prime }S_{h}\left(\zeta \right)I_{m}\left(\zeta \right)\right)d\zeta ,\\ \begin{array}{c} {\;E}_{h}\left(t\right)-{\;E}_{h}\left(0\right)=\frac{1-\tau }{Z\left(\tau \right)}\left(\begin{array}{c} \beta_{h}^{\prime }S_{h}\left(t\right)I_{m}\left(t\right)-\\ \left(\Theta_{H}+\Psi_{H}\right)E_{h}\left(t\right) \end{array}\right)+\frac{\tau }{Z\left(\tau \right)\Gamma \left(\tau \right)}\int_{0}^{t}{\left(t-\zeta \right)}^{\tau -1}\left(\begin{array}{c} \beta_{h}^{\prime }S_{h}\left(\zeta \right)I_{m}\left(\zeta \right)-\\ \left(\Theta_{H}+\Psi_{H}\right)E_{h}\left(\zeta \right) \end{array}\right)d\zeta \\ I_{h}\left(t\right)-I_{h}\left(0\right)=\frac{1-\tau }{Z\left(\tau \right)}\left(\Theta_{H}E_{h}\left(t\right)-\kappa_{H}I_{h}\left(t\right)\right)+\frac{\tau }{Z\left(\tau \right)\Gamma \left(\tau \right)}\int_{0}^{t}{\left(t-\zeta \right)}^{\tau -1}\left(\Theta_{H}E_{h}\left(\zeta \right)-\kappa_{H}I_{h}\left(\zeta \right)\right)d\zeta \\ E_{m}\left(t\right)-E_{m}\left(0\right)=\frac{1-\tau }{Z\left(\tau \right)}\left(\begin{array}{c} \beta_{M}\left(1-E_{m}\left(t\right)-I_{m}\left(t\right)\right)\\ -\left(\Theta_{M}+\Psi_{M}\right)E_{m}\left(t\right) \end{array}\right)+\frac{\tau }{Z\left(\tau \right)\Gamma \left(\tau \right)}\int_{0}^{t}{\left(t-\zeta \right)}^{\tau -1}\left(\begin{array}{c} \beta_{M}\left(1-E_{m}\left(\zeta \right)-I_{m}\left(\zeta \right)\right)\\ -\left(\Theta_{M}+\Psi_{M}\right)E_{m}\left(\zeta \right) \end{array}\right)d\zeta \end{array}\\ I_{m}\left(t\right)-I_{m}\left(0\right)=\frac{1-\tau }{Z\left(\tau \right)}\left(\Theta_{M}E_{m}\left(t\right)-\Psi_{M}I_{m}\left(t\right)\right)+\frac{\tau }{Z\left(\tau \right)\Gamma \left(\tau \right)}\int_{0}^{t}{\left(t-\zeta \right)}^{\tau -1}\left(\Theta_{M}E_{m}\left(\zeta \right)-\Psi_{M}I_{m}\left(\zeta \right)\right)d\zeta .\; \end{array}\right.$$

Let the kernels of system (9) be defined as:

(10) $$\left\{ \begin{array}{c} K_{1}=\Lambda_{H}{\;E}_{h}\left(t\right)+\left(\Lambda_{H}+\varphi \right)\left(1-S_{h}\left(t\right)-E_{h}\left(t\right)-I_{h}\left(t\right)\right)-\beta_{h}^{\prime }S_{h}{\left(t\right)I}_{m}\left(t\right),\\ \begin{array}{c} K_{2}=\beta_{h}^{\prime }S_{h}I_{m}-\left(\Theta_{H}+\Psi_{H}\right)E_{h},\\ K_{3}=\Theta_{H}E_{h}-\kappa_{H}I_{h},\\ K_{4}=\beta_{M}\left(1-E_{m}-I_{m}\right)-\left(\Theta_{M}+\Psi_{M}\right)E_{m}, \end{array}\\ K_{5}=\Theta_{M}E_{m}-\Psi_{M}I_{m}.\; \end{array}\right.$$

Theorem 1 *The kernels at*Eq. (10)*satisfy Lipschitz condition*, $0\leq \eta_{i}<1,i=1\text{,}\ldots ,5$.

***Proof***. *Consider first equation from*(10)*with functions*$S_{h}$*and*$S_{h,1}$:

$$\left\|K_{1}\left(t,S_{h}\right)-K_{1}\left(t,S_{h,1}\right)\right\|=\left\|\left(\Lambda_{H}{\;E}_{h}\left(t\right)+\left(\Lambda_{H}+\varphi \right)\left(1-S_{h}\left(t\right)-E_{h}\left(t\right)-I_{h}\left(t\right)\right)-\beta_{h}^{\prime }S_{h}{\left(t\right)I}_{m}\left(t\right)\right)-\left(\Lambda_{H}{\;E}_{h}\left(t\right)+\left(\Lambda_{H}+\varphi \right)\left(1-S_{h,1}\left(t\right)-E_{h}\left(t\right)-I_{h}\left(t\right)\right)-\beta_{h}^{\prime }S_{h,1}{\left(t\right)I}_{m}\left(t\right)\right)\right\|$$

$$=\left\|-\left(\Lambda_{H}+\varphi \right)S_{h}-\beta_{h}^{\prime }S_{h}{\left(t\right)I}_{m}\left(t\right)+\left(\Lambda_{H}+\varphi \right)S_{h,1}+\beta_{h}^{\prime }S_{h,1}{\left(t\right)I}_{m}\left(t\right)\right\|$$

$$=\left\|-\left(\Lambda_{H}+\varphi \right)\left(S_{h}\left(t\right)-S_{h,1}\left(t\right)\right)-\beta_{h}^{\prime }\left(S_{h}\left(t\right)-S_{h,1}\left(t\right)\right)I_{m}\left(t\right)\right\|$$

$$\leq \left(\left(\Lambda_{H}+\varphi \right)+\beta_{h}^{\prime }\left\|I_{m}\left(t\right)\right\|\right)\left\|S_{h}\left(t\right)-S_{h,1}\left(t\right)\right\|$$

$$=\eta_{1}\left\|S_{h}\left(t\right)-S_{h,1}\left(t\right)\right\|\text{,}$$

*where*$\eta_{1}=\left(\left(\Lambda_{H}+\varphi \right)+\beta_{h}^{\prime }e\right)$*and*$\left\|I_{m}\left(t\right)\right\|=e$, *that is*

$$\left\|K_{1}\left(t,S_{h}\right)-K_{1}\left(t,S_{h,1}\right)\right\|\leq \eta_{1}\left\|S_{h}\left(t\right)-S_{h,1}\left(t\right)\right\|\text{.}$$

*which shows that Lipschitz condition is satisfied for*$K_{1}$, *besides*, *if*$0\leq \eta_{1}<1$, *hence it is a contradiction to definition of*$K_{1}$. *Similarly*, *we obtain*

$$\left\{ \begin{array}{c} \left\|K_{2}\left(t,E_{h}\right)-K_{2}\left(t,E_{h,1}\right)\right\|\leq \eta_{2}\left\|E_{h}-E_{h,1}\right\|,\\ \left\|K_{3}\left(t,I_{h}\right)-K_{3}\left(t,I_{h,1}\right)\right\|\leq \eta_{3}\left\|I_{h}-I_{h,1}\right\|,\\ \left\|K_{4}\left(t,E_{m}\right)-K_{4}\left(t,E_{m,1}\right)\right\|\leq \eta_{4}\left\|E_{m}-E_{m,1}\right\|\text{,}\\ \left\|K_{5}\left(t,I_{m}\right)-K_{5}\left(t,I_{m,1}\right)\right\|\leq \eta_{5}\left\|I_{m}-I_{m,1}\right\|.\; \end{array}\right.$$

*Suppose*l=K(m)×m*and*K(m)*is a Banach space of*R→R*on*m*by*$\left\|S_{h},E_{h},I_{h},E_{m},I_{m}\right\|=\left\|S_{h}\right\|+\left\|E_{h}\right\|+\left\|I_{h}\right\|+\left\|E_{m}\right\|+\left\|I_{m}\right\|$, *in which*$\left\|S_{h}\right\|≔\sup\left\{\left|S_{h}\left(t\right)\right|:t\in m\right\}$, $\left\|E_{h}\right\|≔\sup\left\{\left|E_{h}\left(t\right)\right|:t\in m\right\}$, $\left\|I_{h}\right\|≔\sup\left\{\left|I_{h}\left(t\right)\right|:t\in m\right\}$, $\left\|E_{m}\right\|≔\sup\left\{\left|E_{m}\left(t\right)\right|:t\in m\right\}$. Equation (9)*is articulated in Volterra-type integral as*:

(11) $$\left\{ \begin{array}{c} S_{h}\left(t\right)-S_{h}\left(0\right)=\frac{1-\tau }{Z\left(\tau \right)}\left(\Lambda_{H}{\;E}_{h}\left(t\right)+\left(\Lambda_{H}+\varphi \right)\left(1-S_{h}\left(t\right)-E_{h}\left(t\right)-I_{h}\left(t\right)\right)-\beta_{h}^{\prime }S_{h}{\left(t\right)I}_{m}\left(t\right)\right)+\\ \frac{\tau }{Z\left(\tau \right)\Gamma \left(\tau \right)}\int_{0}^{t}{\left(t-\zeta \right)}^{\tau -1}\left(\Lambda_{H}{\;E}_{h}\left(\zeta \right)+\left(\Lambda_{H}+\varphi \right)\left(1-S_{h}\left(\zeta \right)-E_{h}\left(\zeta \right)-I_{h}\left(\zeta \right)\right)-\beta_{h}^{\prime }S_{h}\left(\zeta \right)I_{m}\left(\zeta \right)\right)d\zeta ,\\ \begin{array}{c} {\;E}_{h}\left(t\right)-{\;E}_{h}\left(0\right)=\frac{1-\tau }{Z\left(\tau \right)}\left(\begin{array}{c} \beta_{h}^{\prime }S_{h}\left(t\right)I_{m}\left(t\right)-\\ \left(\Theta_{H}+\Psi_{H}\right)E_{h}\left(t\right) \end{array}\right)+\frac{\tau }{Z\left(\tau \right)\Gamma \left(\tau \right)}\int_{0}^{t}{\left(t-\zeta \right)}^{\tau -1}\left(\begin{array}{c} \beta_{h}^{\prime }S_{h}\left(\zeta \right)I_{m}\left(\zeta \right)-\\ \left(\Theta_{H}+\Psi_{H}\right)E_{h}\left(\zeta \right) \end{array}\right)d\zeta \\ I_{h}\left(t\right)-I_{h}\left(0\right)=\frac{1-\tau }{Z\left(\tau \right)}\left(\Theta_{H}E_{h}\left(t\right)-\kappa_{H}I_{h}\left(t\right)\right)+\frac{\tau }{Z\left(\tau \right)\Gamma \left(\tau \right)}\int_{0}^{t}{\left(t-\zeta \right)}^{\tau -1}\left(\Theta_{H}E_{h}\left(\zeta \right)-\kappa_{H}I_{h}\left(\zeta \right)\right)d\zeta \\ E_{m}\left(t\right)-E_{m}\left(0\right)=\frac{1-\tau }{Z\left(\tau \right)}\left(\begin{array}{c} \beta_{M}\left(1-E_{m}\left(t\right)-I_{m}\left(t\right)\right)\\ -\left(\Theta_{M}+\Psi_{M}\right)E_{m}\left(t\right) \end{array}\right)+\frac{\tau }{Z\left(\tau \right)\Gamma \left(\tau \right)}\int_{0}^{t}{\left(t-\zeta \right)}^{\tau -1}\left(\begin{array}{c} \beta_{M}\left(1-E_{m}\left(\zeta \right)-I_{m}\left(\zeta \right)\right)\\ -\left(\Theta_{M}+\Psi_{M}\right)E_{m}\left(\zeta \right) \end{array}\right)d\zeta \end{array}\\ I_{m}\left(t\right)-I_{m}\left(0\right)=\frac{1-\tau }{Z\left(\tau \right)}\left(\Theta_{M}E_{m}\left(t\right)-\Psi_{M}I_{m}\left(t\right)\right)+\frac{\tau }{Z\left(\tau \right)\Gamma \left(\tau \right)}\int_{0}^{t}{\left(t-\zeta \right)}^{\tau -1}\left(\Theta_{M}E_{m}\left(\zeta \right)-\Psi_{M}I_{m}\left(\zeta \right)\right)d\zeta .\; \end{array}\right.$$

Equation (11) *is*

(12) $$\left\{ \begin{array}{c} S_{h}\left(t\right)-S_{h}\left(0\right)=\frac{1-\tau }{Z\left(\tau \right)}K_{1}\left(t,S_{h}\right)+\frac{\tau }{Z\left(\tau \right)\Gamma \left(\tau \right)}\int_{0}^{t}{\left(t-\zeta \right)}^{\tau -1}K_{1}\left(\zeta ,S_{h}\right)d\zeta ,\\ {\;E}_{h}\left(t\right)-{\;E}_{h}\left(0\right)=\frac{1-\tau }{Z\left(\tau \right)}K_{2}\left(t,{\;E}_{h}\right)+\frac{\tau }{Z\left(\tau \right)\Gamma \left(\tau \right)}\int_{0}^{t}{\left(t-\zeta \right)}^{\tau -1}K_{2}\left(\zeta ,{\;E}_{h}\right)d\zeta \text{,}\\ I_{h}\left(t\right)-I_{h}\left(0\right)=\frac{1-\tau }{Z\left(\tau \right)}K_{3}\left(t,I_{h}\right)+\frac{\tau }{Z\left(\tau \right)\Gamma \left(\tau \right)}\int_{0}^{t}{\left(t-\zeta \right)}^{\tau -1}K_{3}\left(\zeta ,I_{h}\right)d\zeta ,\\ E_{m}\left(t\right)-E_{m}\left(0\right)=\frac{1-\tau }{Z\left(\tau \right)}K_{4}\left(t,E_{m}\right)+\frac{\tau }{Z\left(\tau \right)\Gamma \left(\tau \right)}\int_{0}^{t}{\left(t-\zeta \right)}^{\tau -1}K_{4}\left(\zeta ,E_{m}\right)d\zeta \text{,}\\ I_{m}\left(t\right)-I_{m}\left(0\right)=\frac{1-\tau }{Z\left(\tau \right)}K_{5}\left(t,I_{m}\right)+\frac{\tau }{Z\left(\tau \right)\Gamma \left(\tau \right)}\int_{0}^{t}{\left(t-\zeta \right)}^{\tau -1}K_{5}\left(\zeta ,I_{m}\right)d\zeta .\; \end{array}\right.$$

*The recursive formula of the* Eq. (12) *shall take the subsequent procedure*

$$\left\{ \begin{array}{c} S_{h,n}\left(t\right)=S_{h}\left(0\right)+\frac{1-\tau }{Z\left(\tau \right)}K_{1}\left(t,S_{h,n-1}\right)+\frac{\tau }{Z\left(\tau \right)\Gamma \left(\tau \right)}\int_{0}^{t}{\left(t-\zeta \right)}^{\tau -1}K_{1}\left(\zeta ,S_{h,n-1}\right)d\zeta ,\\ {\;E}_{h,n}\left(t\right)={\;E}_{h}\left(0\right)+\frac{1-\tau }{Z\left(\tau \right)}K_{2}\left(t,{\;E}_{h,n-1}\right)+\frac{\tau }{Z\left(\tau \right)\Gamma \left(\tau \right)}\int_{0}^{t}{\left(t-\zeta \right)}^{\tau -1}K_{2}\left(\zeta ,{\;E}_{h,n-1}\right)d\zeta \text{,}\\ I_{h,n}\left(t\right)=I_{h}\left(0\right)+\frac{1-\tau }{Z\left(\tau \right)}K_{3}\left(t,I_{h,n-1}\right)+\frac{\tau }{Z\left(\tau \right)\Gamma \left(\tau \right)}\int_{0}^{t}{\left(t-\zeta \right)}^{\tau -1}K_{3}\left(\zeta ,I_{h,n-1}\right)d\zeta ,\\ E_{m,n}\left(t\right)=E_{m}\left(0\right)+\frac{1-\tau }{Z\left(\tau \right)}K_{4}\left(t,E_{m,n-1}\right)+\frac{\tau }{Z\left(\tau \right)\Gamma \left(\tau \right)}\int_{0}^{t}{\left(t-\zeta \right)}^{\tau -1}K_{4}\left(\zeta ,E_{m,n-1}\right)d\zeta \text{,}\\ I_{m,n}\left(t\right)=I_{m}\left(0\right)+\frac{1-\tau }{Z\left(\tau \right)}K_{5}\left(t,I_{m,n-1}\right)+\frac{\tau }{Z\left(\tau \right)\Gamma \left(\tau \right)}\int_{0}^{t}{\left(t-\zeta \right)}^{\tau -1}K_{5}\left(\zeta ,I_{m,n-1}\right)d\zeta .\; \end{array}\right.$$

*where* $S_{h}\left(0\right)$, $E_{h}\left(0\right)$, $I_{h}\left(0\right)$, $E_{m}\left(0\right)$ *and* $I_{m}\left(0\right)\geq 0$.

*Hence*, *we get*

$$\left\{ \begin{array}{c} \left\|W_{n,1}\right\|=\left\|S_{h,n}\left(t\right)-S_{h,n-1}\left(t\right)\right\|\leq \\ \frac{\left(-\tau +1\right)\eta_{1}}{Z\left(\tau \right)}\left\|S_{h,n-1}\left(t\right)-S_{h,n-2}\left(t\right)\right\|+\frac{\;\tau \;\eta_{1}}{\Gamma \left(\tau \right)Z\left(\tau \right)}\int_{0}^{t}{\left(t-\zeta \right)}^{\tau -1}\left\|S_{h,n-1}\left(\zeta \right)-S_{h,n-2}\left(\zeta \right)\right\|d\zeta ,\\ \left\|W_{n,2}\right\|=\left\|E_{h,n}\left(t\right)-E_{h,n-1}\left(t\right)\right\|\leq \\ \frac{\left(-\tau +1\right)\eta_{2}}{Z\left(\tau \right)}\left\|E_{h,n-1}\left(t\right)-E_{h,n-2}\left(t\right)\right\|+\frac{\;\tau \;\eta_{2}}{\Gamma \left(\tau \right)Z\left(\tau \right)}\int_{0}^{t}{\left(t-\zeta \right)}^{\tau -1}\left\|E_{h,n-1}\left(\zeta \right)-E_{h,n-2}\left(\zeta \right)\right\|d\zeta ,\\ \left\|W_{n,3}\right\|=\left\|I_{h,n}\left(t\right)-I_{h,n-1}\left(t\right)\right\|\leq \\ \frac{\left(-\tau +1\right)\eta_{2}}{Z\left(\tau \right)}\left\|I_{h,n-1}\left(t\right)-I_{h,n-2}\left(t\right)\right\|+\frac{\;\tau \;\eta_{2}}{\Gamma \left(\tau \right)Z\left(\tau \right)}\int_{0}^{t}{\left(t-\zeta \right)}^{\tau -1}\left\|I_{h,n-1}\left(\zeta \right)-I_{h,n-2}\left(\zeta \right)\right\|d\zeta \text{,}\\ \left\|W_{n,4}\right\|=\left\|E_{m,n}\left(t\right)-E_{m,n-1}\left(t\right)\right\|\leq \\ \frac{\left(-\tau +1\right)\eta_{2}}{Z\left(\tau \right)}\left\|E_{m,n-1}\left(t\right)-E_{m,n-2}\left(t\right)\right\|+\frac{\;\tau \;\eta_{2}}{\Gamma \left(\tau \right)Z\left(\tau \right)}\int_{0}^{t}{\left(t-\zeta \right)}^{\tau -1}\left\|E_{m,n-1}\left(\zeta \right)-E_{m,n-2}\left(\zeta \right)\right\|d\zeta \text{,}\\ \left\|W_{n,5}\right\|=\left\|I_{m,n}\left(t\right)-I_{m,n-1}\left(t\right)\right\|\leq \\ \frac{\left(-\tau +1\right)\eta_{3}}{Z\left(\tau \right)}\left\|I_{m,n-1}\left(t\right)-I_{m,n-2}\left(t\right)\right\|+\frac{{\tau \;\eta }_{3}}{\Gamma \left(\tau \right)Z\left(\tau \right)}\int_{0}^{t}{\left(t-\zeta \right)}^{\tau -1}\left\|I_{m,n-1}\left(\zeta \right)-I_{m,n-2}\left(\zeta \right)\right\|d\zeta . \end{array}\right.$$

or

$$\left\{ \begin{array}{c} \left\|W_{n,1}\right\|\leq \frac{\left(-\tau +1\right)\eta_{1}}{Z\left(\tau \right)}\left\|W_{n-1,1}\left(t\right)\right\|+\frac{\;\tau \;\eta_{1}}{\Gamma \left(\tau \right)Z\left(\tau \right)}\int_{0}^{t}{\left(-\zeta +t\right)}^{\tau -1}\left\|W_{n-1,1}\left(\zeta \right)\right\|d\zeta ,\\ \left\|W_{n,2}\right\|\leq \frac{\left(-\tau +1\right)\eta_{2}}{Z\left(\tau \right)}\left\|W_{n-1,2}\left(t\right)\right\|+\frac{\;\tau \;\eta_{2}}{\Gamma \left(\tau \right)Z\left(\tau \right)}\int_{0}^{t}{\left(-\zeta +t\right)}^{\tau -1}\left\|W_{n-1,2}\left(\zeta \right)\right\|d\zeta ,\\ \left\|W_{n,3}\right\|\leq \frac{\left(-\tau +1\right)\eta_{3}}{Z\left(\tau \right)}\left\|W_{n-1,3}\left(t\right)\right\|+\frac{\;\tau \;\eta_{3}}{\Gamma \left(\tau \right)Z\left(\tau \right)}\int_{0}^{t}{\left(-\zeta +t\right)}^{\tau -1}\left\|W_{n-1,3}\left(\zeta \right)\right\|d\zeta ,\\ \left\|W_{n,4}\right\|\leq \frac{\left(-\tau +1\right)\eta_{4}}{Z\left(\tau \right)}\left\|W_{n-1,4}\left(t\right)\right\|+\frac{\;\tau \;\eta_{4}}{\Gamma \left(\tau \right)Z\left(\tau \right)}\int_{0}^{t}{\left(-\zeta +t\right)}^{\tau -1}\left\|W_{n-1,4}\left(\zeta \right)\right\|d\zeta ,\\ \left\|W_{n,5}\right\|\leq \frac{\left(-\tau +1\right)\eta_{5}}{Z\left(\tau \right)}\left\|W_{n-1,5}\left(t\right)\right\|+\frac{{\tau \;\eta }_{5}}{\Gamma \left(\tau \right)Z\left(\tau \right)}\int_{0}^{t}{\left(-\zeta +t\right)}^{\tau -1}\left\|W_{n-1,5}\left(\zeta \right)\right\|d\zeta .\; \end{array}\right.$$

Theorem 2 *Model (5) has a unique solution if*

$$\frac{1-\tau }{Z\left(\tau \right)}\eta_{i}+\frac{t_{\max}^{\tau +1}}{\Gamma \left(\tau \right)Z\left(\tau \right)}\eta_{i}<1,i=1\text{,}\ldots ,5\text{.}$$

***Proof***. *Suppose*$S_{h}\left(t\right)$*and*$S_{h,1}\left(t\right)$*are solutions*, *and thus*

$$S_{h}\left(t\right)-S_{h,1}\left(t\right)=\frac{1-\tau }{Z\left(\tau \right)}\left(K_{1}\left(t,S_{h}\right)-K_{1}\left(t,S_{h,1}\right)\right)+\frac{\tau }{Z\left(\tau \right)\Gamma \left(\tau \right)}\int_{0}^{t}{\left(t-\zeta \right)}^{\tau -1}\left(K_{1}\left(\zeta ,S_{h}\right)-K_{1}\left(\zeta ,S_{h,1}\right)\right)d\zeta \text{,}$$

$$\left\|S_{h}\left(t\right)-S_{h,1}\left(t\right)\right\|\leq \frac{1-\tau }{Z\left(\tau \right)}\left\|K_{1}\left(t,S_{h}\right)-K_{1}\left(t,S_{h,1}\right)\right\|+\frac{\tau }{Z\left(\tau \right)\Gamma \left(\tau \right)}\int_{0}^{t}{\left(t-\zeta \right)}^{\tau -1}\left\|K_{1}\left(\zeta ,S_{h}\right)-K_{1}\left(\zeta ,S_{h,1}\right)\right\|d\zeta \text{,}$$

$$\left\|S_{h}\left(t\right)-S_{h,1}\left(t\right)\right\|\leq \frac{\left(1-\tau \right)\eta_{1}}{Z\left(\tau \right)}\left\|S_{h}\left(t\right)-S_{h,1}\left(t\right)\right\|+\frac{t^{\tau }\eta_{1}}{Z\left(\tau \right)\Gamma \left(\tau \right)}\left\|S_{h}\left(\zeta \right)-S_{h,1}\left(\zeta \right)\right\|\text{.}$$

*This implies*

$$\left(1-\frac{\left(1-\tau \right)\eta_{1}}{Z\left(\tau \right)}-\frac{t^{\tau }\eta_{1}}{Z\left(\tau \right)\Gamma \left(\tau \right)}\right)\left\|S_{h}\left(t\right)-S_{h,1}\left(t\right)\right\|\leq 0\text{,}$$

which implies that

$$\left\|S_{h}\left(t\right)-S_{h,1}\left(t\right)\right\|=0\text{.}$$

*Therefore*,

$$S_{h}\left(t\right)=S_{h,1}\left(t\right)\text{.}$$

*Hence*, *the model solution is unique*.

## 6 Solutions to Eq. (5)

We present numerical solutions to Eq. (4) by the method at PI [35]. Consider

(13) Dtτ0ABCU(t)=H(t,U(t)),

along with ICs $U\left(t_{0}\right)=U_{0}$, where H(t,U(t)) is continuous. Applying the integral operator, we get from (13):

(14) $$U\left(t\right)-U\left(0\right)=\frac{1-\tau }{Z\left(\tau \right)}H\left(t,U\left(t\right)\right)+\frac{\tau }{\Gamma \left(\tau \right)Z\left(\tau \right)}\int_{0}^{t}{\left(t-\zeta \right)}^{\tau -1}H\left(\zeta ,U\left(\zeta \right)\right)d\zeta \text{.}$$

Taking $t=t_{n}=t_{0}+n\;h$ in (14), where the step size is h, we attain

(15) $$U\left(t_{n}\right)=U\left(t_{0}\right)+\frac{1-\tau }{Z\left(\tau \right)}H\left(t_{n},U\left(t_{n}\right))+t_{n}\right))+t_{n},U\frac{\tau }{\Gamma \left(\tau \right)Z\left(\tau \right)}\sum_{t=0}^{n-1}\int_{t_{0}}^{t}{\left(t_{n}-\zeta \right)}^{\tau -1}H\left(\zeta ,U\left(\zeta \right)\right)d\zeta \text{.}$$

Now, one can estimate the function H(ζ,U(ζ)) with the help of first order Lagrange interpolation,

(16) $$H\left(\zeta ,U\left(\zeta \right)\right)\approx H\left(t_{i+1},U_{i+1}\right)+\frac{\zeta -t_{i+1}}{h}\left(H\left(t_{i+1},U_{i+1}\right)-H\left(t_{i},U_{i}\right)\right),\zeta \in \left[t_{i},t_{i+1}\right]\text{,}$$

in which $U_{i}=U\left(t_{i}\right)$. Writing (15) in (16) and by ABC product-integration (ABCPI) accomplished [36], we obtain:

(17) $$U_{n}=U_{0}+\frac{\tau \;h^{\tau }}{Z\left(\tau \right)}\left(\;A_{n}H\left(t_{0},U_{0}\right)+\sum_{i=1}^{n}B_{n-i}\;H\left(t_{i},U_{i}\right)\right),n\geq 1$$

where

(18) $$\left\{ \begin{array}{c} A_{n}=\frac{{\left(n-1\right)}^{\tau }-n^{\tau }\left(n-\tau -1\right)}{\Gamma \left(\tau +2\right)},\\ B_{j}=\left\{ \begin{array}{c} \frac{1}{\Gamma \left(\tau +2\right)}+\frac{1-\tau }{\tau \;h^{\tau }}\;j=0,\\ \frac{{\left(j-1\right)}^{\tau +1}-{2j}^{\tau +1}+{\left(1+j\right)}^{\tau +1}}{\Gamma \left(\tau +2\right)},j=1,2,...n-1. \end{array}\right. \end{array}\right.$$

Rate of convergence of ABCPI method is τ+1, i.e., $\left|U\left(t_{n}\right)-U_{n}\right|=O\left(h^{1+\tau }\right)$ [[36], [37], [38]]. Computational cost is proportionate to $O\left(N\;log_{2}\left(N\right)\right)$ relative to $O\left(N^{2}\right)$ as in any other prevalent discretization algorithm (see Refs. [[39], [40], [41], [42], [43], [44]]).

## 7 Application of the ABC-PI method to Eq. (5)

By applying the algorithm described at 17) and (18) to system (5), we get:

$$\left\{ \begin{array}{c} S_{h,n}=S_{h,0}+\frac{\tau \;h^{\tau }}{Z\left(\tau \right)}\left(\begin{array}{c} A_{n}\left(\Lambda_{H}{\;E}_{h,0}+\left(\Lambda_{H}+\varphi \right)\left(1-S_{h,0}-E_{h,0}-I_{h,0}\right)-\beta_{h}^{\prime }S_{h,0}I_{m,0}\right)+\\ \sum_{i=1}^{n}B_{n-i}\;\left(\Lambda_{H}{\;E}_{h,i}+\left(\Lambda_{H}+\varphi \right)\left(1-S_{h,i}-E_{h,i}-I_{h,i}\right)-\beta_{h}^{\prime }S_{h,i}I_{m,i}\right) \end{array}\right),\\ E_{h,n}=E_{h,0}+\frac{\tau \;h^{\tau }}{Z\left(\tau \right)}\left(\begin{array}{c} A_{n}\left(\beta_{h}^{\prime }S_{h,0}I_{m,0}-\left(\Theta_{H}+\Psi_{H}\right)E_{h,0}\right)+\\ \sum_{i=1}^{n}B_{n-i}\;\left(\beta_{h}^{\prime }S_{h,i}I_{m,i}-\left(\Theta_{H}+\Psi_{H}\right)E_{h,i}\right) \end{array}\right),\\ I_{h,n}=I_{h,0}+\frac{\tau \;h^{\tau }}{Z\left(\tau \right)}\left(\begin{array}{c} A_{n}\left(\Theta_{H}E_{h,0}-\kappa_{H}I_{h,0}\right)+\\ \sum_{i=1}^{n}B_{n-i}\;\left(\Theta_{H}E_{h,i}-\kappa_{H}I_{h,i}\right) \end{array}\right),\\ E_{m,n}=E_{m,0}+\frac{\tau \;h^{\tau }}{Z\left(\tau \right)}\left(\begin{array}{c} A_{n}\left(\beta_{M}\left(1-E_{m,0}-I_{m,0}\right)-\left(\Theta_{M}+\Psi_{M}\right)E_{m,0}\right)+\\ \sum_{i=1}^{n}B_{n-i}\;\beta_{M}\left(1-E_{m,i}-I_{m,i}\right)-\left(\Theta_{M}+\Psi_{M}\right)E_{m,i} \end{array}\right),\\ I_{m,n}=I_{m,0}+\frac{\tau \;h^{\tau }}{Z\left(\tau \right)}\left(\begin{array}{c} A_{n}\left(\Theta_{M}E_{m,0}-\Psi_{M}I_{m,0}\right)+\\ \sum_{i=1}^{n}B_{n-i}\;\left(\Theta_{M}E_{m,i}-\Psi_{M}I_{m,i}\right) \end{array}\right).\; \end{array}\right.$$

## 8 Sensitivity analysis

Sensitive analysis helps us to see the variation in the variables when changing the parameters occurring in the $R_{0}$. It tells us which component of $R_{0}$ play a vital role in the model under observed.

**Definition 1** [34]**.** *Normalized forward sensitivity index of* $R_{0}$ *is:*

$$H_{\mu }=\left(\frac{\partial R_{0}}{\partial \mu }\right)\left(\frac{\mu }{R_{0}}\right)\text{,}$$

To determine sensitivity indices, techniques namely (a) linearization method, (b) Latin hypercube sampling (c) direct differentiation method can be used and then solving the consequences obtained from any technique. Over here category (c) is used. The indices help us which indices have positive influence and which have negative impact. It is also useful for generating strategies to control disease.

**Fig. 2:**
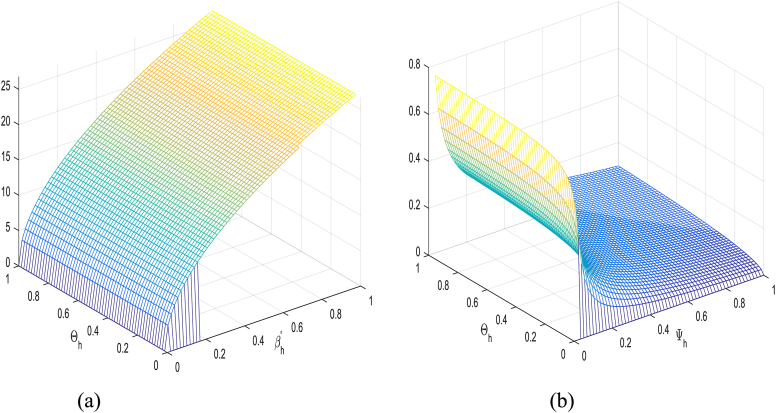
**(a)** The analysis of $R_{0}$ against $\beta_{h}^{\prime }$ and $\Theta_{H}$ and (b) The analysis of $R_{0}$ against $\Psi_{h}$ and $\Theta_{H}$.

**Fig. 3:**
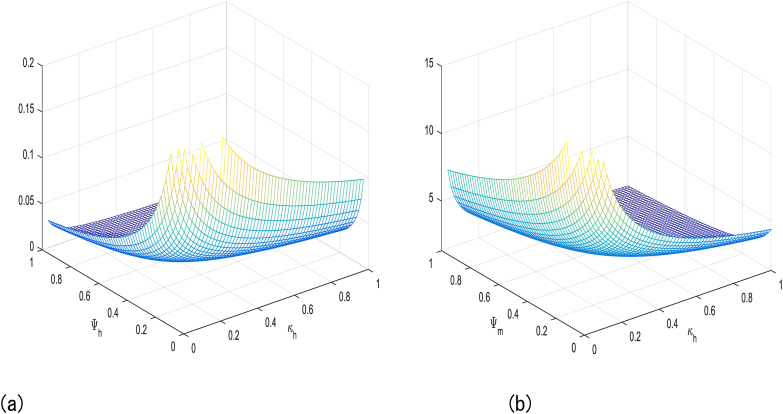
We show graphical results of sensitivity analysis of $R_{0}$ against the given parameters: (a)The analysis of $R_{0}$ against $\kappa_{H}$ and $\Psi_{h}$ (b) The analysis of $R_{0}$ against $\kappa_{H}$ and $\Psi_{M}$.

**Fig. 4:**
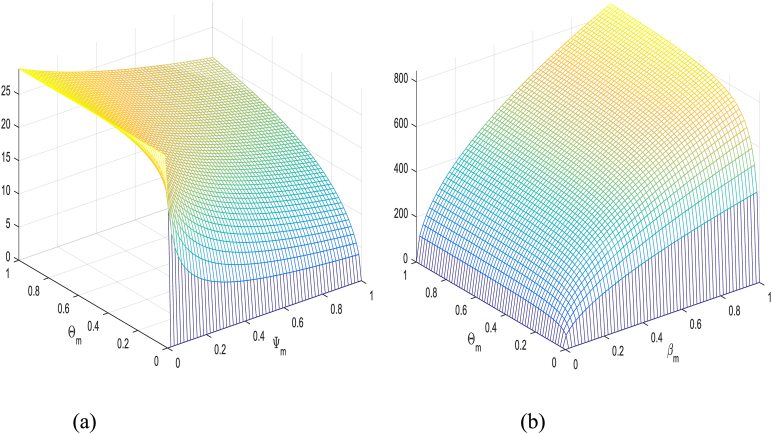
We show graphical results of sensitivity analysis of $R_{0}$ against the given parameters: (a) The analysis of $R_{0}$ against $\Psi_{M}$ and $\Theta_{M}$ (b) The analysis of $R_{0}$ against $\beta_{M}$ and $\Theta_{M}$.

**Fig. 5:**
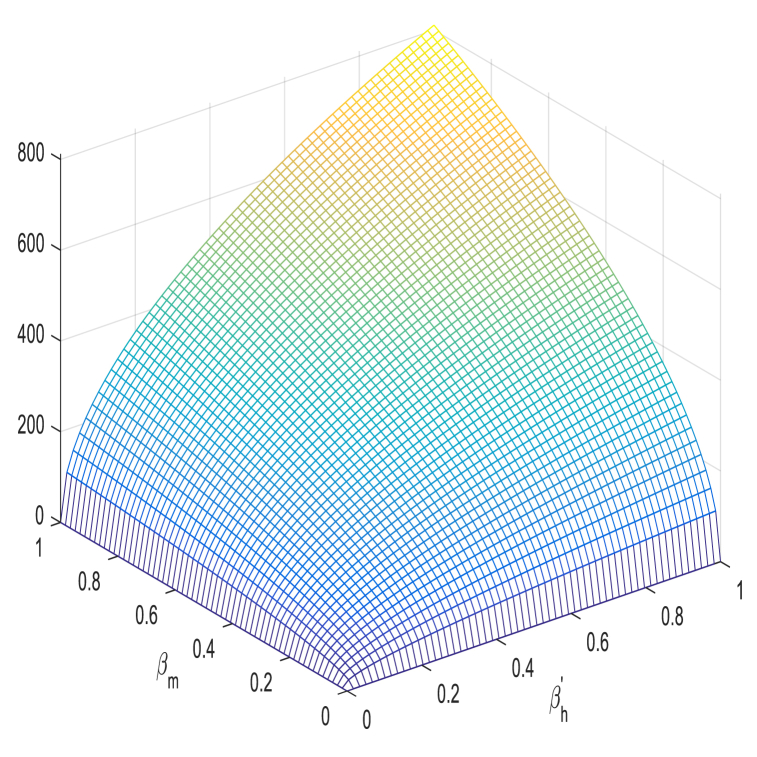
The analysis of $R_{0}$ against $\beta_{h}^{\prime }$ and $\beta_{M}\text{.}$.

$\beta_{h}^{\prime }$, $\Theta_{H}$, $\beta_{M}$, and $\Theta_{M}$ in Table 1 have a positive impact on $R_{0}$, which refer to that the decay or growth of these strictures, e.g., 10 % shall surge or decline $R_{0}$ by 5 %, 0.03 %, 5 %, and 2 % respectively. However contrariwise, the index for strictures $\Psi_{h}$, $\Psi_{M}$ and $\kappa_{H}$ demonstrates increasing their value by 10 % shall drop value of $R_{0}$ by 0.03 %, 5 % and 7 % respectively. Fig. 2, Fig. 3, Fig. 4, Fig. 5 shows sensitivity analysis of different parameters with $R_{0}$. Fig. 2 with subfigures (a) and (b) show respectively the analysis of $R_{0}$ against $\beta_{h}^{\prime }$ and $\Theta_{H}$ and $R_{0}$ against $\Psi_{h}$ and $\Theta_{H}$. Similarly, we have other parameters of the model that influences the basic reproduction number have been represents graphically in Fig. 3, Fig. 4, Fig. 5.

**Table 1:** Sensitivity indices of $R_{0}$.

Parameter	H. Index	Value	Parameter	H. Index	Value
$\beta_{h}^{\prime }$	$H_{\beta_{h}^{\prime }}$	0.5000000	$\kappa_{H}$	$H_{\kappa_{H}}$	−0.5000000
$\Theta_{H}$	$H_{\Theta_{H}}$	0.0002912	$\Psi_{M}$	$H_{\Psi_{M}}$	−0.6999999
$\Psi_{h}$	$H_{\Psi_{h}}$	−0.0002912	$\Theta_{M}$	$H_{\Theta_{M}}$	0.2000000
$\beta_{M}$	$H_{\beta_{M}}$	0.4999999

## 9 Discussion and outcomes

We used the notations: $\Lambda_{H}=1/25$ days, $\beta_{h}^{\prime }=0.6989\;$ days, $\Theta_{H}=0.068630$ days, $\Psi_{H}=0.00004$ days, $\kappa_{H}=0.0228$ days, φ=0.054 days, $\Psi_{M}=1/15$ days, $\Theta_{M}=1/10$ days, $\beta_{M}=0.001088$ days, $S_{h}\left(0\right)=1$, $E_{h}\left(0\right)=0.1$, $I_{h}\left(0\right)=0.1$, $E_{m}\left(0\right)=0.01$ and $I_{m}\left(0\right)=0.1$.

**Fig. 6:**
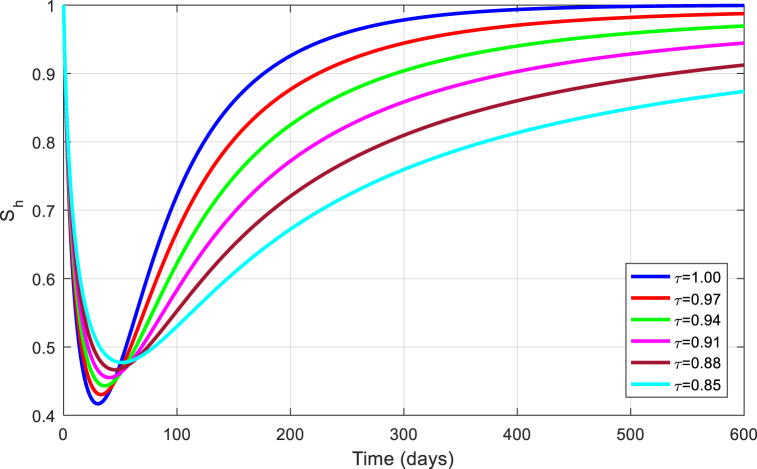
Simulation of $S_{h}$ for DFE with τ.

**Fig. 7:**
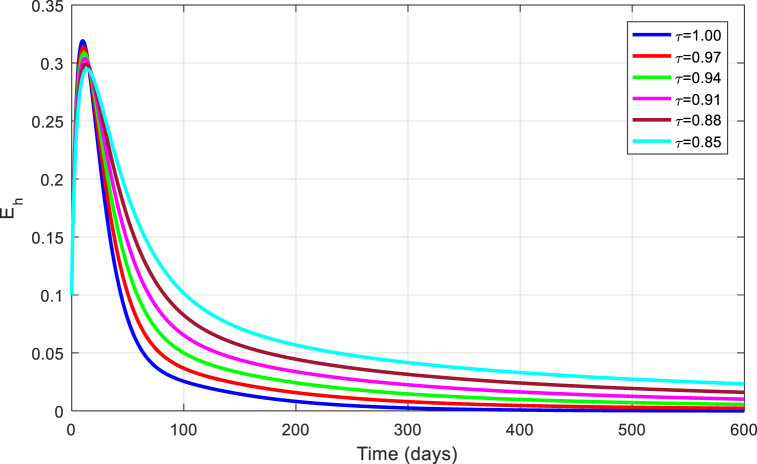
Simulation of $E_{h}$ for DFE with τ.

**Fig. 8:**
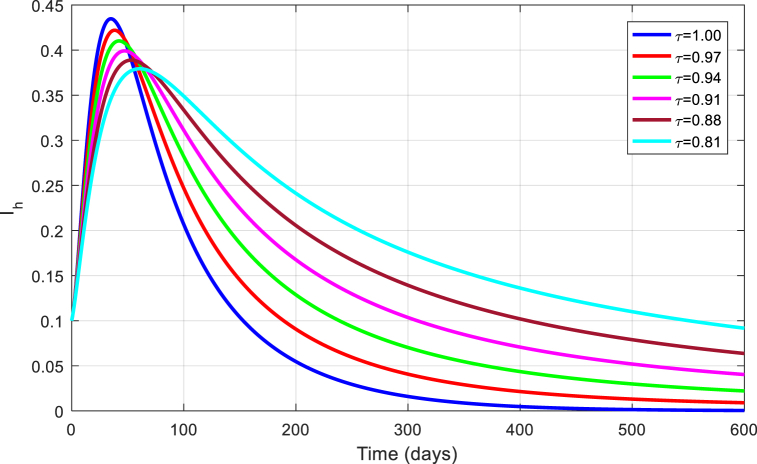
Simulation of $I_{h}$ for DFE with τ.

**Fig. 9:**
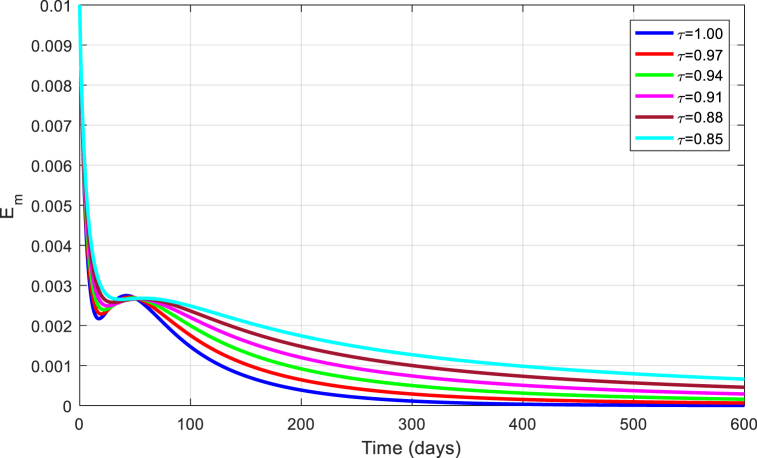
Simulation of $E_{m}$ for DFE with τ.

**Fig. 10:**
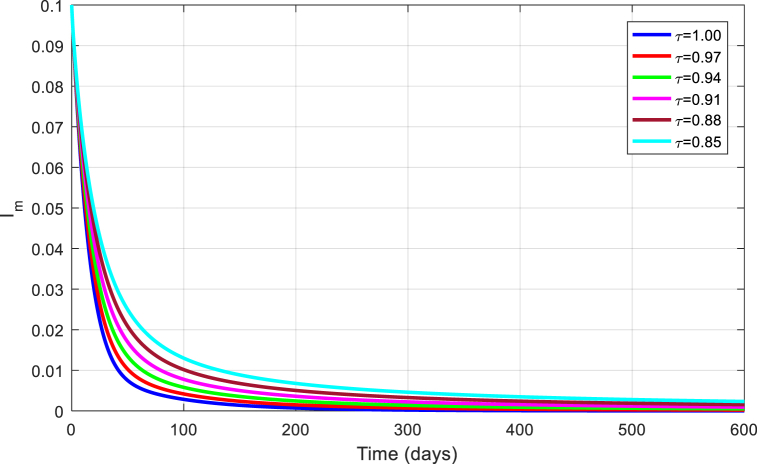
Simulation of $I_{m}$ for DFE with τ.

The simulations are seen in Fig. 6, Fig. 7, Fig. 8, Fig. 9, Fig. 10 for different values of τ for the case of the DFE. If we take $\beta_{M}=0.01088$ and the rest of values given above, the condition for stability of the equilibrium point $E_{1}$ will be provided. The simulations are clearly visible in Fig. 6, Fig. 7, Fig. 8, Fig. 9, Fig. 10 for different values of τ. When τ=1, the simulations of ZVM for endemic and disease-free equilibrium points are almost the same as in Ref. [1]. The DFE equilibrium when using arbitrary values assigned to the fractional order τ have been represented by Fig. 6, Fig. 7, Fig. 8, Fig. 9, Fig. 10 for the DFE case. We see that the equilibrium DFE is locally asymptotically stable for various values of the order τ..

**Fig. 11:**
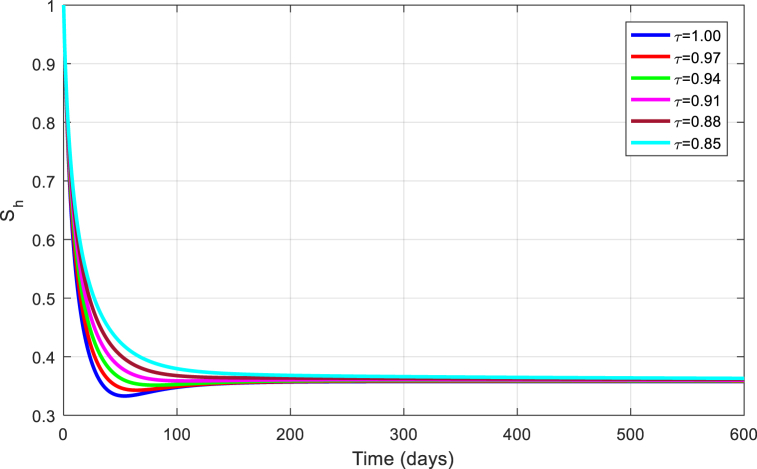
Simulation of $S_{h}$ for EE with τ.

**Fig. 12:**
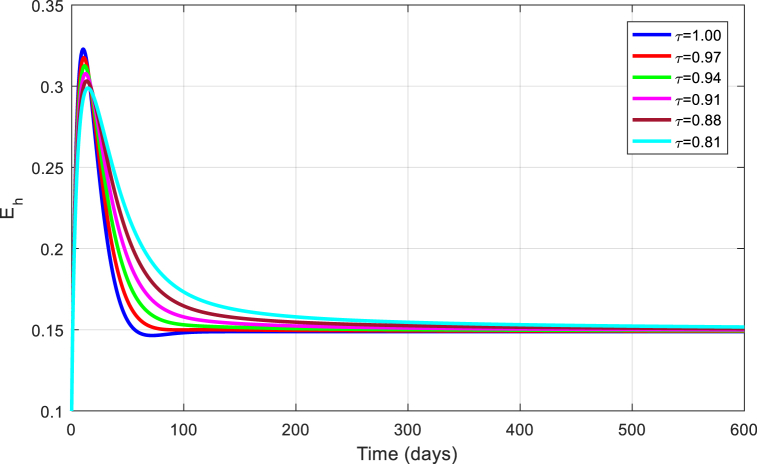
Simulation of $E_{h}$ for EE with τ.

**Fig. 13:**
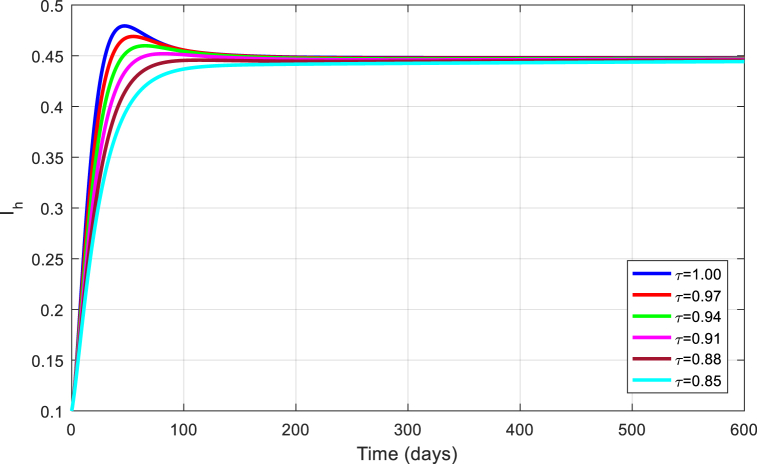
Simulation of $I_{h}$ for EE with τ.

**Fig. 14:**
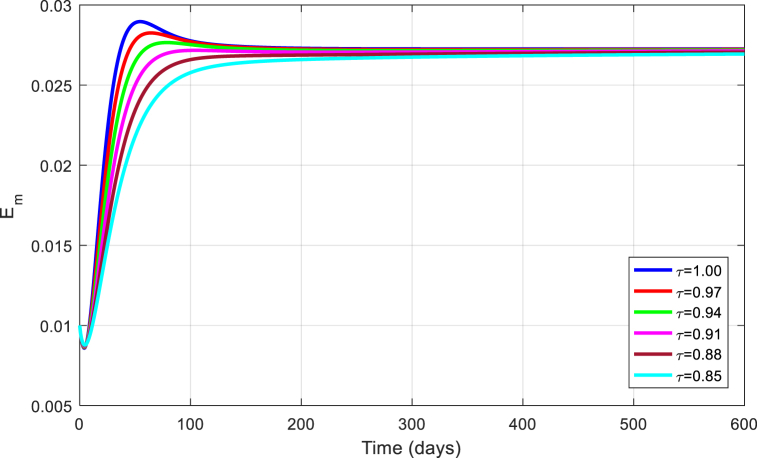
Simulation of $E_{m}$ for EE with τ.

**Fig. 15:**
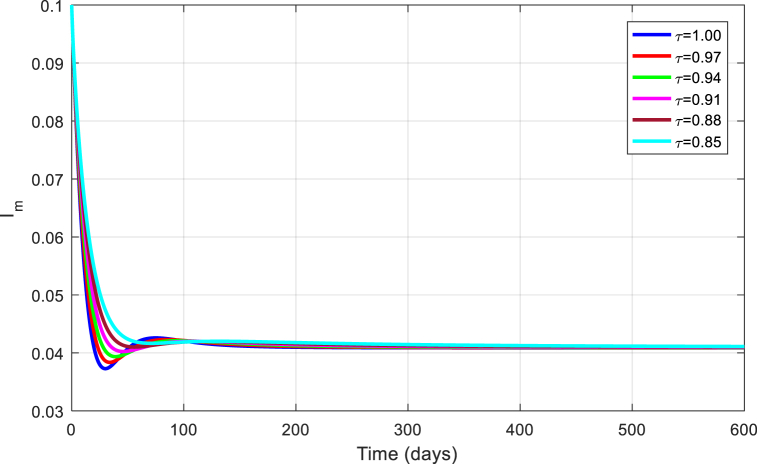
Simulation of $I_{m}$ for EE with τ.

Fig. 11, Fig. 12, Fig. 13, Fig. 14, Fig. 15 have been obtained using the EE by using different values of the order τ. We see when changing the order τ, the EE approaches to the EE endemic equilibrium.

The disease epidemic modeling and their analysis is more complex, so it is necessary to analyze the model, when the model under consideration is complex. To capture more complexities of our model, we used the fractional differentiation and integration to better analyze the complexities involve the ZIKA virus model with the observed real data.

## 10 Conclusion

In this paper, an iterative technique is considered, namely, the ABCPI method to analyze Zika system. We fitted the ZV data to the model using the parameters values suggested in Ref. [1], and it can be seen that the work presented in Ref. [1] for the data fitting when changing the value of $\beta_{h}=0.5689$, for τ=1, we have good results with $R_{0}=0.4942$. We studied graphically the equilibrium points, disease free and endemic case. Numerical solution converges to the disease-free equilibrium and endemic case. This analysis suggests that the numerical scheme with PI is good for the approximation of nonlinear biological system with real data and provide better results.

### Data availability statement

Data is available online from Reference [9], which can be accessed through the following link.

https://www.sciencedirect.com/science/article/abs/pii/S0378475419300679.

## Additional information

No additional information is available for this paper.

## CRediT authorship contribution statement

**Zain Ul Abadin Zafar:** Writing - review & editing, Writing - original draft, Methodology, Data curation, Conceptualization. **Muhammad Altaf Khan:** Writing - review & editing, Writing - original draft, Validation, Software, Resources, Investigation, Data curation, Conceptualization. **Mustafa Inc:** Writing - review & editing, Writing - original draft, Visualization, Supervision, Methodology, Conceptualization. **Ali Akgül:** Writing - review & editing, Writing - original draft, Validation, Formal analysis, Data curation. **Mohammed Asiri:** Writing - review & editing, Validation, Supervision, Formal analysis, Data curation. **Muhammad Bilal Riaz:** Writing - review & editing, Visualization, Validation, Methodology, Investigation, Data curation.

## Declaration of competing interest

The authors declare that they have no conflict of interest.
